# Effect of Off-Pump Coronary Artery Bypass Surgery on Patients’ Quality of Life

**DOI:** 10.14740/cr326e

**Published:** 2014-02-27

**Authors:** Mohammadhosain Afrand, Seyed Khalil Froozan-Nia, Hamide Dehghani, Mehrdad Jalalian, Mohammadtaghi Sarebanhassanabadi

**Affiliations:** a Medical Scientific Association,Yazd Cardiovascular Research Center, Shahid Sadoughi University of Medical Sciences, Yazd, Iran; b Department of Cardiac Surgery, Yazd Cardiovascular Research Center, Shahid Sadoughi University of Medical Sciences, Yazd, Iran; c Department of Nursing and Midwifery, Yazd Cardiovascular Research Center, Shahid Sadoughi University of Medical Sciences, Yazd, Iran; d Editor In-Chief, Electronic Physician, Mashhad, Iran; e Yazd Cardiovascular Research Center, Shahid Sadoughi University of Medical Sciences, Yazd, Iran

**Keywords:** Quality of life, Off-pump coronary artery bypass, Nottingham Health Profile

## Abstract

**Background:**

Off-pump coronary artery bypass (OPCAB) surgery is a common technique used to control the incidence of myocardial ischemia and to increase the lifespan of patients with coronary artery disease (CAD). There is still considerable controversy about effect of OPCAB on patients’ quality of life. The purpose of this study was to determine the effect of OPCAB on different aspects of the patients’ quality of life.

**Methods:**

A total of 190 patients who underwent elective OPCAB surgery at Afshar Hospital in Yazd, Iran, from October 2012 through April 2013 participated in the study. Nottingham Health Profile (NHP) questionnaire was used in structured interviews before and 3 months after the OPCAB surgery. Independent samples t-test, Mann-Whitney test and analysis of variance were used for statistical analysis.

**Results:**

Quality of life from the aspect of pain (P = 0.014), energy (P = 0.001), emotional reaction (P < 0.001), social isolation (P = 0.002) and physical activity (P = 0.005) were significantly different, but there was no significant difference in sleep between men and women (P = 0.06). Women achieved a better quality of life 3 months after OPCAB surgery (P < 0.001). There was no significant difference in patients’ quality of life in terms of their ejection fraction (P = 0.06). There was no significant difference in patients’ quality of life in terms of their New York Heart Association (NYHA) functional class score (P = 0.57). Patients’ quality of life scores before OPCAB surgery and 3 months after the surgery showed no significant difference after adjusting for history of myocardial infarction (P = 0.82), hyperlipoproteinemia (P = 0.38), cigarette addiction (P = 0.2), hypertension (P = 0.7) and diabetes mellitus (P = 0.15).

**Conclusion:**

Most aspects of patients’ quality of life were better after OPCAB surgery. The most obvious finding to emerge from this study was that women’s quality of life was better than men’s after OPCAB surgery. Since CAD is prevalent and OPCAB is one way to treat and manage this disease, patients’ quality of life can be improved if they are managed appropriately after OPCAB surgery (especially for men).

## Introduction

Coronary artery disease (CAD) is defined as the narrowing or blockage of all or part of the coronary artery lumen due to the process of atherosclerosis, spasm and thrombosis. Often in CAD, the diseased artery cannot provide the oxygen required by the myocardial muscle, which usually leads to angina and myocardial infarction. CAD was the leading cause of sudden death and disability from 1950 to 2000 throughout the world [1-3]. In many cases, coronary artery bypass graft (CABG) surgery is the only way to control the incidence of myocardial ischemia and angina attacks and extend the lifespan of these patients [3, 4]. Off-pump coronary artery bypass (OPCAB) surgery is a type of minimally invasive, cardiac surgery in which cardiopulmonary bypass is eliminated and the heart continues to beat [5]. CABG without the use of cardiopulmonary bypass (off-pump bypass) is performed in order to reduce the post-operative complications associated with the use of cardiopulmonary bypass, including generalized systemic inflammatory response, cerebral dysfunction, myocardial depression and hemodynamic instability [6].

Many studies have been conducted on the efficacy of CABG in the treatment of CAD. Formerly, the results from this type of treatment were evaluated mainly in the form of mortality, using measures such as survival, laboratory data and clinical judgment [7]. Although these methods provide very important information regarding the patient’s body, they are not able to assess other aspects of the patient’s health. Thus, in case of progressive and chronic diseases, such as coronary heart disease, where the disease cannot be separated from the social structure and person’s personality, the stated criteria will not suffice to investigate all aspects of the patient’s health. Thus, the concept of quality of life is used to determine the patient’s social and personal background. Also, valuable information that may be ignored by the doctor can be acquired by using this measure [8]. Currently, due to the development of modern techniques and improvements in the management of patients after surgery, the prognosis has improved for patients who must undergo coronary artery bypass surgery [9].

One of the most important methods is OPCAB surgery, which is considered to be a physiological technique when compared with conventional methods in which a pump is used (on-pump CAB) [10]. Quality of life is a multi-dimensional concept that is related to all physical, psychological and social aspects of health. Studies have shown that, after the surgery, patients experience various physical, emotional and social problems and they perceive that their quality of life has decreased. Lukkarinen et al indicated that women with CAD had a lower perception of their quality of life than men with CAD had. The quality of life for women with CAD was lower than the quality of life for healthy women in the same age group. For women with CAD, their emotional reactions and social isolation were closely correlated with their demographic characteristics, such as smoking, economic status, depression and life’s unpleasant experiences [11]. Sjoland et al studied the improvement of quality of life and the difference in quality of life between men and women 3 months, 1 year and 2 years after CABG surgery. All aspects of quality of life improved in both genders after CABG surgery. Although men had better physical abilities and overall health than women, ultimately the women’s quality of life was better than that of the men after surgery [12].

Studies have shown significant benefits of OPCAB surgery over conventional CABG surgery with cardiopulmonary bypass for mortality [13], morbidity [14], length of stay and cost [15]. Researchers and clinicians increasingly are recognizing the importance of assessing a wide range of outcomes when evaluating the efficacy of medical therapies or procedures [16]. Despite several studies on patients’ quality of life before and after OPCAB surgery, there is no consensus on what is a good time to assess quality of life in patients who have undergone CABG surgery [17]. According to the concept of quality of life and due to the problems that CAD patients are facing before and after treatment, rehabilitation of these patients is of great importance. This study aimed to compare patients’ quality of life and all of its aspects before and after OPCAB surgery.

## Materials and Methods

This was a cross-sectional study that included 190 patients who underwent elective OPCAB surgery at Afshar Hospital in Yazd, Iran, from October 2012 through April 2013. If patients met the inclusion criteria, they were selected by a simple, randomized method. The size of the sample was determined by the following formula:

n = Z^2^ × P × (1-p)/(c)^2^ = (1.96)^2^ × 0.5 × (1-0.5)/(0.07)^2 ^= 195

where n is the sample size, Z is the Z value (for example, 1.96 for 95% confidence level), p is the percentage picking a choice, expressed as a decimal, P = 0.5, for determining the largest sample size needed and C is the confidence interval, expressed as a decimal.

Inclusion and exclusion criteria of the study were considered in two steps. Research baseline criteria (24 h before OPCAB surgery) included endemic candidates for OPCAB surgery, patients who were hospitalized for OPCAB surgery for the first time and patients were in the age range of 35-85. Non-native patients, those with significant handicaps or disabilities, and patients who had experienced severe stressful events, such as divorce, a relative’s death, or financial failure during the preceding 6 weeks were excluded. Exclusion criteria 3 months after OPCAB surgery were second cardiac and non-cardiac surgery; having experienced severe physical or emotional stress, such as the death of a spouse or a close relative; serious illness; and the patient’s death from any cause during OPCAB surgery and before completing the second-stage form. The quality of life analysis was performed using the Nottingham Health Profile (NHP) questionnaire part 1 [18], the efficiency of which has been confirmed many times in clinical studies [19].

The study of Nehrir et al confirmed the test-retest reliability of the questionnaire (r = 0.87) [20]. In another study, Dehdari et al used test-retest to confirm the reliability of the questionnaire, and the correlation reliability was measured as 85% [21]. The original questionnaire was written in English, but it was translated into Persian, and its linguistic accuracy was validated. The NHP questionnaire even deals well with the circumstance in which older people disregard the actual state of their health and report it as being “good”. The questionnaire achieves this by focusing on the discomfort and pain that people feel in addition to their stated opinions of the state of their health [22]. Further, the statements are related to problems rather than to symptoms, as is often the case with most other self-evaluation instruments. As a result, there is a lower probability that patients will be able to “medicalize” their psychological and social stress. The NHP questionnaire part 1 contains 38 subjective statements divided into six sections, namely physical activity, social isolation, emotional reaction, energy, pain and sleep. The scores of each section range from 0 to 100, and they are determined by adding the items’ weight, based on the Turnstone method of paired comparisons, to every positive answer [23]. A higher score indicates a higher level of dysfunction and worse quality of life. The ejection fraction (EF) was identified by echocardiography using the Simpson method.

After the surgery, the patients were in the intensive care unit, where they were monitored carefully using a constant electrocardiogram, as well as respiratory monitoring. The patients were observed and assessed carefully to identify the presence and the absence of post-operative complications. We distributed the questionnaire to all patients before their OPCAB surgeries and 3 months after their OPCAB surgeries. Demographic data were collected from medical records, medical offices, and reception and discharge records. Three months after the OPCAB surgery, a form and detailed instructions for completing the form were sent to the patients. However, before that was done, the patients were asked if they would be willing to come to the Afshar Cardiac Clinic on the due date to complete the questionnaire. If the patients did not fill out the first form completely or did not return it at all, another form was sent to them. Patients who did not complete the questionnaire properly even after 1 month of follow-up phone calls were excluded from the study. Having obtained approvals from hospital authorities and the head of the ward, the researcher was admitted to the hospital and received the patients’ written informed consent forms in which they acknowledged that they understood what the study entailed and agreed to participate. Patients were informed that they could withdraw from the study whenever they wished and that they would be excluded if they chose not to cooperate with the researchers. They were assured that their therapeutic relationship with their physician and the hospital would not be affected in either case. The patients also were assured that their information would be kept secret.

The data are presented as mean ± standard deviation (SD). The unpaired t-test was used to examine the differences in the two genders. The pre-operative quality of life and the post-operative quality of life for the two genders were assessed by the Mann-Whitney test. The differences in various aspects of their quality of life (physical activity, social isolation, emotional reaction, energy, pain and sleep) were examined by the analysis of variance test. We compared pre-operative and post-operative results of every quality of life section with reference values, which were obtained by means examinations of the general population [22], taking into account the genders and the age distribution of the patients. The differences between pre-operative and post-operative NYHA class score, EF and the coronary vessels that were involved were examined by the Mann-Whitney test. All statistical analyses were performed using SPSS software (version 17; SPSS, Chicago, IL, USA). All statistical tests were two-sided, and differences with probability values < 0.05 were considered to be statistically significant.

## Results

The pre-operative sample size was 210. Twelve patients chose not to have the OPCAB surgery, and eight patients who had major surgery, such as heart valve replacements, were excluded, leaving a total of 190 patients who participated in the study, of whom 141 were males (74.3%) and 49 were females (25.7%). The mean age of the participants was 58.7 ± 11, and 94.3% were married, and 49.4% had a primary school education. The majority of the patients had EFs in the 30 to 49% range, while 56.8% had NYHA functional class score ІІІ or ІV, and 69.5% had three vessels involved. Evaluation of risk of ischemic disease showed that 53.5% had reported a history of hypertension, 53.9% had hyperlipoproteinemia, 34.9% had diabetes mellitus, 31.7% were addicted to cigarettes and 37.9% had experienced a myocardial infarction. Quality of life scores before the OPCAB surgery and 3 months after the surgery showed no significant difference from the results of the Wilcoxon test (P ∼ 0.495), for which lower scores indicate a better quality of life (Table 1). The NHP quality of life scores before the OPCAB surgery and 3 months afterwards in both men and women showed that there were significant differences in the quality of life scores from the aspects of pain (P = 0.014), energy (P = 0.001), emotional reaction (P < 0.001), social isolation (P = 0.002) and physical activity (P = 0.005); however, there was no significant difference in sleep (P = 0.06) between men and women. (Note that lower scores indicate better quality of life.) Women had lower quality of life before the OPCAB surgery, but they had better quality of life 3 months after the surgery (P < 0.001) (Table 2).

**Table 1:** Comparison of Pre-Operative and Post-Operative Quality of Life Scores

uality of life	Post-operation Mean ± SD	Pre-operation Mean ± SD	P
Energy	2.02 ± 0.98	2.05 ± 0.97	0.85
Pain	3.21 ± 2.64	3.20 ± 2.64	0.96
Emotional reaction	4.07 ± 2.60	4.10 ± 2.60	0.87
Sleep	1.46 ± 1.81	2.61 ± 1.46	0.98
Social isolation	1.46 ± 1.81	1.47 ± 1.54	0.8
Physical activity	3.50 ± 1.90	3.44 ± 1.90	0.68
Total	17.95 ± 8.77	17.58 ± 8.84	0.49

**Table 2:** Comparison of the Mean Change of the Pre-Operative and Post-Operative Quality of Life Scores By Gender and Age Group

Quality of life			Pre-operation Mean ± SD	Post-operation Mean ± SD	Mean change Pre- and post-operation	P
Aspects	Compared variable					
Energy	Gender	Male	1.91 ± 0.97	2.06 ± 0.99	0.15 ± 1.29	0.001
		Female	2.55 ± 0.75	1.92 ± 0.98	-0.58 ± 1.16	
	Age group (years)	< 50	2.36 ± 0.85	1.95 ± 1.03	-0.4 ± 1.1	0.02
		> 50	1.93 ± 0.99	2.02 ± 0.98	0.12 ± 3. 6	
Pain	Gender	Male	2.94 ± 2.58	3.27 ± 2.72	0.394 ± 3.53	0.014
		Female	4.06 ± 2.74	2.92 ± 2.37	-1.15 ± 3.77	
	Age group (years)	< 50	4.17 ± 2.67	2.36 ± 2.19	-1.8 ± 3.09	> 0.001
		> 50	2.79 ± 2.53	3.41 ± 2.69	0.63 ± 2.60	
Emotional reaction	Gender	Male	3.73 ± 2.53	4.26 ± 2.63	0.54 ± 3.48	> 0.001
		Female	5.36 ± 2.43	3.55 ± 2.47	-1.89 ± 3.7	
	Age group (years)	< 50	4.63 ± 2.51	3.32 ± 2.56	-1.44 ± 2.89	0.004
		> 50	3.90 ± 2.61	4.28 ± 2.54	3.82 ± 0.38	
Sleep	Gender	Male	2.6 ± 1.49	2.76 ± 1.48	0.18 ± 2.15	0.06
		Female	2.66 ± 1.44	2.19 ± 1.46	-0.49 ± 1.75	
	Age group (years)	< 50	2.68 ± 1.49	2.49 ± 1.35	2.04 ± 0.17	0.52
		> 50	2.58 ± 1.47	2.65 ± 1.53	-0.06 ± 2.11	
Social isolation	Gender	Male	1.23 ± 1.42	1.45 ± 1.45	0.22 ± 1.94	0.002
		Female	2.25 ± 1.65	1.44 ± 1.55	-0.87 ± 2.41	
	Age group (years)	< 50	1.73 ± 1.4	1.19 ± 1.39	-0.61 ± 1.52	0.032
		> 50	1.34 ± 1.56	1.51 ± 1.49	0.15 ± 2.23	
Physical activity	Gender	Male	3.25 ± 1.83	3.5 ± 1.95	0.39 ± 2.6	0.005
		Female	4.15 ± 1.93	3.36 ± 1. 7	-0.91 ± 2.57	
	Age group (years)	< 50	3.69 ± 1.99	3.02 ± 1.92	0.63 ± 2.47	0.039
		> 50	3.33 ± 1.86	3.61 ± 1.85	0.31 ± 2.75	
Total	Gender	Male	16.4 ± 8.52	18.56 ± 8.96	2.64 ± 11.78	> 0.001
		Female	21.75 ± 8.52	16.12 ± 8.19	-5.53 ± 12	
	Age group (years)	< 50	20.06 ± 8.46	15.59 ± 8.56	-4.64 ± 9.5	0.003
		> 50	16.5 ± 8.2	18.44 ± 8.6	2.22 ± 12.79	

After the quality of life scores before and after the OPCAB surgery were adjusted to account for age, significant differences were observed for the aspects of pain (P < 0.001), energy (P = 0.02), emotional reaction (P = 0.004), social isolation (P = 0.032) and physical activity (P = 0.039); again sleep (P = 0.52) was the exception. If the mean age was less than 50, a better quality of life was achieved after the OPCAB surgery (Table 2). Quality of life scores before the OPCAB surgery and 3 months afterwards showed no significant difference after adjustments were made for education level (P ∼ 0.27) and marital status (P ∼ 0.83).

There were no significant differences in the quality of life scores in terms of their EF (P = 0.06). There were no significant differences in the quality of life scores in terms of their NYHA class score (P = 0.57). Patients’ quality of life after making adjustments for the number of coronary vessels involved indicated that patients with one vessel coronary involved had higher scores for quality of life after the OPCAB surgery, but the difference was not significant (P ∼ 0.79). The quality of life scores before the OPCAB surgery and 3 months afterwards showed no significant differences after adjustments were made for the following: history of myocardial infarction (P = 0.82), hyperlipoproteinemia (P = 0.38), cigarette addiction (P = 0.2), hypertension (P = 0.7) and diabetes mellitus (P = 0.15) (Table 3).

**Table 3:** Comparison of the Pre-Operative and Post-Operative Quality of Life Scores By Patients’ History of Risk of Ischemic Disease

Quality of life		Pre-operation Mean ± SD	Post-operation Mean ± SD	Mean change	P
Ischemic disease risk history					
MI	Yes	17.92 ± 8.45	18.97 ± 8.75	-0.9 ± 12.08	0.82
	No	17.4 ± 9.24	17.45 ± 8.86	0.44 ± 12. 32	
HLP	Yes	18.75 ± 9.33	19.30 ± 9.87	0.42 ± 12.91	0.38
	No	16.38 ± 8.26	16.67 ± 7.47	0.69 ± 11.67	
Cigarette addiction	Yes	19.47 ± 7.24	19.17 ± 8.32	2.48 ± 10.9	0.2
	No	18.14 ± 9.53	17.41 ± 8.76	-0.24 ± 12.8	
HTN	Yes	18.04 ± 9.52	18.59 ± 8.91	0.7 ± 12.63	0.7
	No	17.15 ± 8.05	17.16 ± 8.77	0.41 ± 12.02	
DM	Yes	17.51 ± 10.11	19.78 ± 9.22	2.77 ± 13.94	0.15
	No	17.62 ± 8.17	17.07 ± 8.48	0.31 ± 11.48	

MI: myocardial infarction; HLP: hyperlipoproteinemia; HTN: hypertension; DM: diabetes mellitus.

## Discussion

The results indicated that the average quality of life scores before the OPCAB surgery and 3 months afterwards had no statistically significant differences. Scores for most of the aspects of quality of life increased after the surgery, including those for physical activity, social isolation, emotional reaction, energy and pain. Sjoland and Meyler studied the scores for various aspects of the quality of life before the OPCAB surgery, 3 months afterwards and 6 months afterwards. They concluded that, 3 months after surgery, the perceived quality of life was lower than it was before the surgery and 6 months after the surgery. Post-operative complications, such as chest pain, difficulty sleeping due to pain and shortness of breath, can affect patients’ quality of life for a long time, preventing them from returning to their normal activities until they completely recover from the surgery [12, 24]. However, the results of research studies vary, and assessments of quality of life should be conducted 6 to 12 months after surgery to allow for complete recovery. El Baz et al believed that bypass surgery does not solely improve quality of life, because the patients’ varying abilities to control psychological problems and stress have an important role as well [25]. However, according to Mercier, patients’ quality of life is low before the surgery. After CABG surgery, quality of life improves at first due to relief from pain and the psychological support that is received, but, over time and with improving physical activities, their quality of life increases [26]. Covinsky’s study also stated that the quality of life in post-menopausal women was significantly different 6 months and 1 year after coronary surgery, and the patients achieved a better quality of life over time [27].

Brorsson et al believed that patients’ quality of life gradually increases for a few years after surgery. He also noted that appropriate follow-up, controlling any ensuing illnesses and receiving attention from family members can contribute to increased quality of life in the long term. They believed that patients’ anxiety about potential or actual surgical complications and about the costs involved result in a lower quality of life in the short term [1]. Obviously, the results of this study were consistent with some of the results of prior studies, for example that the quality of life is low in the first months after the OPCAB surgery and that it increases in the long term. Another important finding was that women have better quality of life than men after OPCAB surgery, whereas just the opposite was true before the surgery. But, after the OPCAB surgery, the scores indicated that several aspects of quality of life were improved, namely energy, pain, emotional, social and physical activity, resulting in a significant difference.

In a study by Peric entitled “Quality of Life in Men and Women before and after Bypass Surgery”, based on the NHP scores, women had higher scores for quality of life before CABG that means their quality of life was lower than men (Note that lower scores indicate better quality of life.). But the quality of life after CABG was lower than that of men [28]. Most reports were about the worse pre-operative clinical assessment of women than men, namely women had a severe degree of coronary disease just before CABG [12, 29]. Ayanian et al studied the physical and psycho-social functioning of women and men after CABG. Women were more seriously ill at the time of the operation than men were. Although post-operative depression occurred in both men and women, it occurred more often in women (up to 60%). However, 6 months after CABG, there was a similar rate of psychological recovery in both genders [30]. In this study, educational levels showed no significant difference on their effects on quality of life before and after CABG. A previous study indicated that patients’ quality of life was significantly different in the general health of physical component domains of Short Form 36 (SF-36) items. Conversely, patients who had university education possessed the highest scores, followed by those with primary education and secondary education, in that order [31].

The result of this study showed no significant difference in terms of NYHA class score before the OPCAB surgery and 3 months afterwards in both men and women. Peric et al showed that, 6 months after CABG, the NYHA function class score was improved significantly in both men and women. However, women had higher symptomatology than men before and after the CABG surgery, and their results were worse than their NYHA class score indicated. Also, the women were older and had a larger number of associated diseases before the CABG, facts that should be taken into account when making comparative analyses [12].

Generally, our results showed that women have better quality of life after OPCAB surgery than men do, which was consistent with Chocron and Fayyazi’s findings [27, 32, 33]. Quality of life scores after adjusting for age group showed that, after OPCAB surgery, the quality of life was higher for patients whose ages were less than 50. Increasing age can be one of the risk factors of OPCAB surgery. The outcomes and quality of life values are inversely related to the age at which the surgery was performed. This is understandable because increasing age is accompanied by physical, psychological and social problems as well as pain. Hose et al found no significant relationship between age and quality of life after CABG [31]. One of the limitations of the current study is its cross-sectional nature. In studies such as ours, causal relationships cannot be demonstrated exactly. Therefore, in order to show the patients’ quality of life after surgery, prospective longitudinal studies are needed.

### Conclusions

In summary, patients’ quality of life was better after OPCAB surgery in its most aspects, including physical activity, social isolation, emotional reaction, energy and pain. The most obvious finding to emerge from this study was that women’s quality of life was better than that of men after OPCAB surgery, whereas just the opposite was true before surgery. However, we were unable to respond completely to the question that which aspects of the patients’ lives were most affected, that is, whether it was energy, pain, emotional reactions, sleep, social isolation or physical activity. It is recommended that future studies investigate the quality of life 6 months to 1 year after OPCAB surgery. Further studies of patients’ undergoing elective OPCAB surgery using the cohort methodology could be a good direction for future research on this issue.
